# Semi-automatic determination of elemental sulphur in rubber

**DOI:** 10.1155/S1463924697000151

**Published:** 1997

**Authors:** K. Saraswathi, K. Vijayalakshmi, P. Prameela

**Affiliations:** Department of Chemistry Sri Venkateswara University Tirupati 517 502 India

## Abstract

Electro-active elemental sulphur dissolved in hydrazine hydrate solvent was studied by d.c. polarography, cyclic voltammetry and millicoulometry in sodium acetate, ammonium tartrate and sodium phosphate buffers in an aqueous medium. The method, which uses a mercury electrode, is highly sensitive with less interference than other polarographic methods. The method was extended to the determination of sulphur in pressure rubber tubing. This paper also suggests a mechanism of electrode reaction.


Journal of Automatic Chemistry, Vol. 19, No. 5 (September–October 1997) pp. 153–156

### Semi-automatic determination of elemental sulphur in rubber

**K. Saraswathi, K. Vijayalakshmi and P. Prameela**

*Department of Chemistry, Sri Venkateswara University, Tirupati, 517 502, India*

*Electro-active elemental sulphur dissolved in hydrazine hydrate solvent was studied by d.c. polarography, cyclic voltammetry and millicoulometry in sodium acetate, ammonium tartrate and sodium phosphate buffers in an aqueous medium. The method, which uses a mercury electrode, is highly sensitive with less interference than other polarographic methods. The method was extended to the determination of sulphur in pressure rubber tubing. This paper also suggests a mechanism of electrode reaction.*

### Introduction

Application of polarography in the petroleum industry to determine various forms of sulphur began in the 1950s and since then several procedures have been developed, which have widened the application to other fields [1]. A.C. polarography is considered to be a promising method for detecting micro quantities of sulphur in crude oil, petrochemicals and ointments [2]. Volkova and Doronova [3] determined the sulphur present in vulcanization accelerators with an accuracy of ±5% and sensitivity of 0·005%.

A two-electron cathodic reduction of sulphur was suggested in N,N-dimethyl acetamide and N,N-dimethyl formamide by Rotinyan *et al.* [4]. Oscillation of instantaneous current of sulphur by oscillopolarography has been attributed to changes in thickness and tearing of non-conducting film formed by the interaction of mercury with sulphur [5].

Sulphur present in ilmenite, niobium and indium phosphides has been determined by differential pulse polarography [6]. A two electron reduction to $S^{2-}$ in aluminium chloride–sodium chloride melts was proposed by Paulsen *et al.* [7]. Using an oxygen flask combustion method, Al-Abachi *et al.* [8] reported indirect polarographic determination of sulphur in organic compounds and drugs. Employing the same method, simultaneous determination of sulphur and phosphorous in organic compounds has been made possible by Bishara and El-Samman [9]. In order to determine the sulphur content in the Elbe River in different seasons, the sediments were extracted with toluene and the sulphur was determined by differential pulse voltammetry on an SMDE in a mixed toluene–methanol/ammonium acetate–acetic acid electrolyte by Kirste *et al.* [10].

In these methods quantitative analysis of sulphur was attempted using different organic solvents. But there were problems with the quantitative analysis if any interfering sulphur containing vulcanization accelerators dissolved in organic solvents was present. These are reduced at the potential nearly coinciding with the reduction potentials of sulphur. To minimize these interferences, a method has been developed using a solvent, hydrazine hydrate in sodium hydrogen phosphate plus sodium nitrate, sodium acetate and ammonium tartrate supporting electrolytes.

### Experimental

Commercial sulphur was purified by repeated recrystallization from carbon disulphide. As sulphur is insoluble in water, hydrazine hydrate was used as the solvent. Supporting electrolytes used were 0·1M ammonium tartrate + 0·1M potassium nitrate (pH 8·5 to 12·0), 0·1M sodium acetate + sodium hydroxide (Walpole acetate buffer pH 9·0 to 11·0) and 0·1M sodium hydrogen phosphate + 0·1M sodium nitrate (pH 9·5 to 12·0). The pH of the solutions was adjusted using alkalis or acids with common cations.

The polarograms were recorded with an ELICO recording polarograph—Model CL-25, Brinkmann Bank-Elektronic instrument—for cyclic voltammetric recordings and a millicoulometer supplied by Radelkis (Budapest) for coulometry. pH measurements were made with a pH meter, model L1-10 from ELICO (Pvt) Ltd, India.

### Results and discussion

### *D.C. polarographic results*

Sulphur gave a well-defined single anodic wave in acetate and tartrate supporting electrolytes in 4–8% aqueous hydrazine hydrate when pH ranged from 9·0 to 12·0 (see figures 1 and 2). In a phosphate medium a composite wave was observed under the same conditions (see figure 3). The anodic wave can be attributed to the oxidation of mercury by the sulphide ions present in the solution which were formed by the dissolution of sulphur in hydrazine hydrate. A composite wave of sulphur in the phosphate medium was due to elemental sulphur and sulphide ions being present in the solution.

With increasing pH, the half-wave potentials ($E\frac{1}{2}$ = −0·685 V versus SCE in pH 9·0, −0·710 V versus SCE in pH 10 and −0·750 V versus SCE in pH 11) shifted to more negative values in all three supporting electrolytes indicating proton involvement in the electrode reaction. The limiting current remained constant with pH in the corresponding supporting electrolyte. At pH 10·0 and above a characteristic minima was observed at more negative potentials on the limiting current plateau; this is common when sulphur and sulphide ions are both presented in solution (see figure 4). A sodium acetate medium of pH 9·0 was selected for a detailed study as

0142-0453/97 $12·00 © 1997 Taylor & Francis Ltd

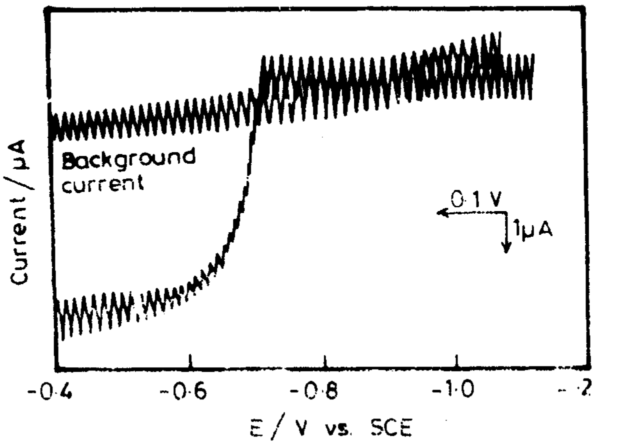

*Figure 1. Polarogram: 0·1M sodium acetate and sodium hydroxide (Walpole acetate buffer).*

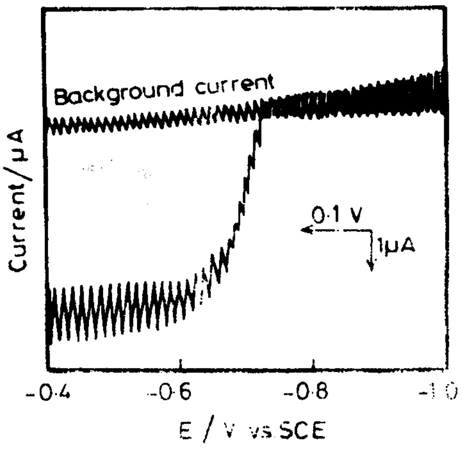

*Figure 2. Polarogram: 0·1M ammonium tartrate, 0·1M potassium nitrate.*

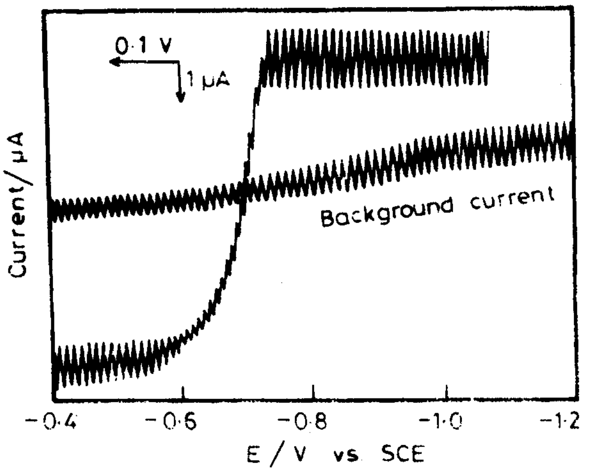

*Figure 3. Polarogram: 0·1M sodium hydrogen phosphate and 0·1M sodium nitrate supporting electrolyte.*

both minima and composite waves were absent. A simple method was developed for the determination of sulphur in rubber samples. The shape and height of the sulphur polarograms remain constant with varying concentrations of the components of the supporting electrolytes, in the range of 0·02M to 1·0M.

A linear plot of $i_d$ versus $C$ in the concentration range of 0·1 to 1·5 mM sulphur passing through the origin in a sodium acetate supporting electrolyte at pH 9·0 indicates the diffusion-controlled nature of the wave in that medium and the validity of the method for the quantitative estimation of sulphur. The diffusion current constant values are found to be 3·0 and, applying the Lingane equation, $n$ corresponded to 2·0. At a fixed pH with increasing sulphur concentration, there was no shift in

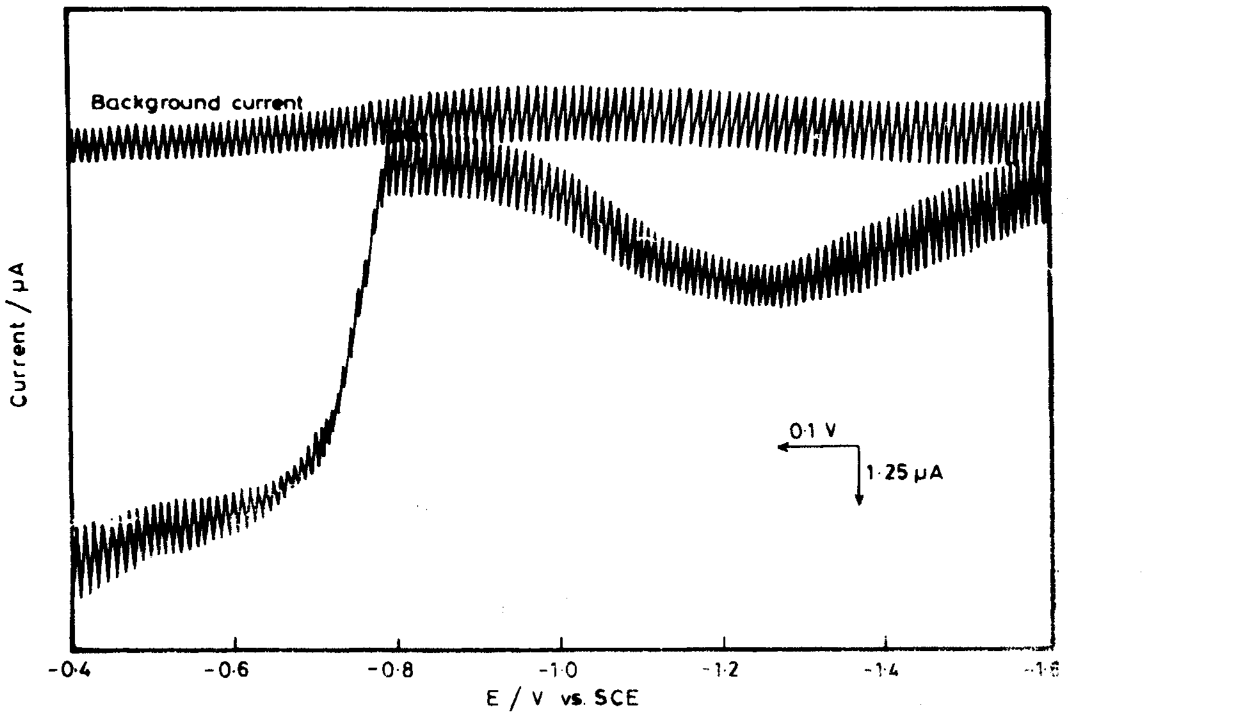

*Figure 4. Typical polarogram of sulphur in alkaline medium.*

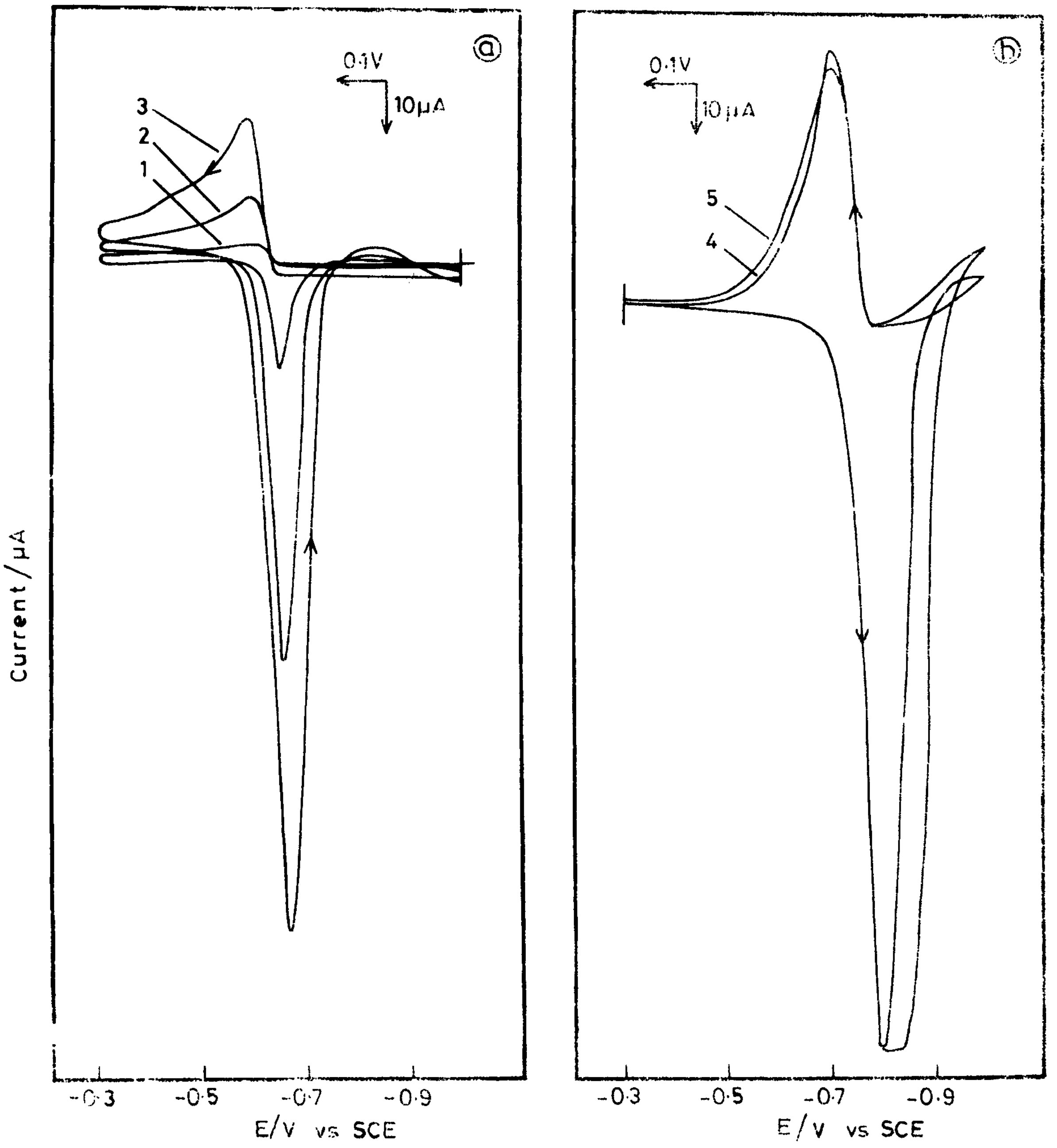

*Figure 5. Multicyclic voltammograms of sulphur in 4% hydrazine hydrate.*

half-wave potential which also indicates reversibility of the electrode process. The surface active material, TX-100, had no influence on the characteristic minima at higher pHs. In 10% hydrazine hydrate, and in an acetate-supporting electrolyte, a single anodic–cathodic wave was observed whose half-wave potential coincides with 4% and 8% hydrazine hydrate values. The waves were found to be diffusion controlled from the constant values of $i_d/\sqrt{h}$.

### Cyclic voltammetric results

A cyclic voltammogram on sulphur in 4–8% hydrazine hydrate in an acetate-supporting electrolyte, at pH 9·0 in the scan range −0·3 V to −1.0 V versus SCE, is shown in figure 5. Analogous to polarographic data, a single anodic wave at −0·7 V is seen. The peak potential difference $(Ep, a/Ep, c)$ is around 30–35 mV, indicating a two-electron oxidation process. The anodic as well as cathodic peak potentials are constant with the change in concentration of sulphur and scan rates, which indicates the reversible nature of the electrode reaction.

The electrode reaction is not a diffusion-controlled process in C.V., either at very low scan rates (0·05 to 0·08 $Vs^{-1}$) or in sweep rates. The current function $ip$ versus $V^{1/2}$ also deviates from linearity at very low scan rates. Multicycle voltammograms confirm this as the peak currents continue to rise until the third or fourth cycle for both anodic and cathodic peaks (figure 5). The adsorbed film can be attributed to the oxidation of

*Table 1. Cyclic voltammetric data (sulphur in 4% hydrazine hydrate).*

Sulphur concentration : 0·5 mM
Supporting electrolyte : 0·1 M sodium acetate + sodium hydroxide
pH ~9·0

| Sample No. | Sweep rate $V/Vs^{-1}$ | Ep,a V (vs SCE) | Ip,a μA | Ip,a* $V^{1/2}C$ | Ep,c V (vs SCE) | Ip,c μA | Ip,c* $V^{1/2}C$ |
|---|---|---|---|---|---|---|---|
| 1 | 0·04 | −0·700 | 2·50 | 17·85 | −0·76 | 21·0 | 150 |
| 2 | 0·06 | −0·700 | 3·00 | 17·85 | −0·75 | 24·5 | 145 |
| 3 | 0·08 | −0·700 | 5·75 | 29·30 | −0·75 | 42·0 | 214 |
| 4 | 0·10 | −0·700 | 6·50 | 29·90 | −0·75 | 50·5 | 232 |
| 5 | 0·20 | −0·700 | 9·00 | 29·20 | −0·75 | 71·5 | 232 |
| 6 | 0·40 | −0·685 | 11·00 | 24·90 | −0·75 | 60·0 | 138 |

* Units: $\mu A\, S^{1/2}\, V^{-1/2}\, mol^{-1}\, l$.

*Table 2. Determination of sulphur in rubber samples.*

| Sample No. | Sample weight (g) | Amount of sulphur found: Present method* (g) | Amount of sulphur found: Proske method (g) |
|---|---|---|---|
| 1 | 0.50 | 0·192 | 0·191 |
| 2 | 0·49 | 0·188 | 0·187 |

* Average of 10 individual determinations.

mercury in the presence of sulphide in the first anodic scan. This gets reduced in the cathodic scan, forming hydrogen sulphide. This further enhances the oxidation of mercury in the succeeding multiple cycles thus increasing both the peak currents (anodic and cathodic) in multicyclic voltammograms (see table 1).

*Coulometric results*

Millicoulometry of sulphur in 8% hydrazine hydrate solution using an acetate supporting electrolyte of pH 9·0 at −0·5 V shows a two-electron oxidation over a mercury pool electrode. The electrolysis is complete in a short time.

### Conclusions

The anodic waves observed in 4–8% hydrazine hydrate can be attributed to oxidation of mercury by the sulphide formed as a result of the dissolution of sulphur in hydrazine hydrate.

$$Hg + S^{2-} \Longleftrightarrow HgS + 2H^+ + 2e^-$$

The D.C. polarographic, cyclic voltammetric and coulometric data account for this mechanism which involves two protons and two electrons and a reversible process. The mercuric sulphide is reduced to hydrogen sulphide, which results in the cathodic peak in cyclic voltammetry. Therefore, the reaction is:

$$HgS + 2e^- + 2H^+ \rightarrow Hg + H_2S$$

The composite wave obtained with higher hydrazine hydrate concentrations may be attributed to the presence of sulphide and sulphur together in the alkaline solution. The equilibrium present in the solution may be represented by the equation:

$$S + S^{2-} \leftrightarrow S_2^{2-}$$

The characteristic minima observed in 10% hydrazine hydrate in a sodium acetate supporting electrolyte may be due to the formation of disulphide ions.

*Application to rubber samples*

Anodic diffusion controlled polarograms of sulphur dissolved in hydrazine hydrate (4–8%) were used in this study for determining the amount of sulphur present in rubber samples. The sample was extracted into acetone by digesting the solution for four hours in a Soxhlet extraction apparatus. Acetone from the extract was removed by distillation and the elemental sulphur left behind was dissolved in hydrazine hydrate and its polarogram was recorded in acetate buffer. The results were checked with 10 duplicate experiments and were found to be reproducible (SD 0·0005), and accurate when compared to the Proske method [11] (see table 2). These results show that the method described is highly sensitive; it also requires less time and has fewer interferences than the methods previously reported for determining the amount of elemental sulphur in rubber samples.

### References

1. *Encyclopedia of Electrochemistry of the Elements* (1973), 292.
2. Khim Joon Yong, Chang and Soon Ja, Y., *Akhak. Hoechi*, **22,** 4 (1978), 201.
3. Volkova, G. G. and Doronova, A. M., *Khim. Prom-St-Ser., Metody Anal, Kontrolya Kack. Prod. Khim. Prom-Sti*, **7** (1979), 16.
4. Rotinyan, A. L., Shishkina, S. V. and Tikhonov, K. I., *Electrokhimiya*, **10,** 4 (1974), 444.
5. Fleszar, B., Kowalski, J. and Bieniasz, H., *Rocz. Chem.*, **47,** 6 (1973), 1287.
6. Kaplan Ya, B., Varavko, T. N. and Vladimirskaya, I. N., *Zavod. Lab.*, **44,** 3 (1978), 268.
7. Paulsen, K. A., Osteryoung, A. and Robert, A., *Journal of the American Chemical Society*, **98,** 22 (1976), 6866.
8. Al-Abaghi, Moudyed, Q., At Dabbagh, Fawzi, H. and Sulaiman, S. T., *Talanta*, **27,** 12 (1980), 1077.
9. Bishara, S. B. and El-Samman, F. M., *Microchemical Journal*, **27,** 1 (1982), 44.
10. Kirste, S., Moeller, A., Helms, I., Scholz, F., Clans, E. and Heininger, P., *Fresenius Environ. Bull.*, **4,** 3 (1995), 143–147.
11. Proske, G. E. O., *Angew. Chem.*, **A59** (1947), 121.

